# Improving the management of non-ST elevation acute coronary syndromes: systematic evaluation of a quality improvement programme *E*uropean *QU*ality *I*mprovement *P*rogramme for *A*cute *C*oronary *S*yndrome: The EQUIP-ACS project protocol and design

**DOI:** 10.1186/1745-6215-11-5

**Published:** 2010-01-14

**Authors:** Marcus D Flather, Jean Booth, Daphne Babalis, Hector Bueno, Philippe G Steg, Grzegorz Opolski, Filippo Ottani, Jacques Machecourt, Alfredo Bardaji, Mats Bojestig, Anthony R Brady, Bertil Lindahl

**Affiliations:** 1 Clinical Trials and Evaluation Unit, Royal Brompton Hospital, Sydney Street, SW7 6NP, UK; 2 Hospital General Universitario "Gregorio Marañón", Dr Esquerdo 46, 28007 Madrid Spain; 3 INSERM U-698, Université Paris 7 and Assistance Publique-Hôpitaux de Paris, Paris, France; 4 Warszawski Uniwersytet Medyczny, Żwirki i Wigury 61, 02-091, Warszawa, Poland; 5 Ospedale Morgagni Pierantoni, Via Forlanini 34, 47100 Forli, Italy; 6 Centre Hospitalo-Universitaire Grenoble, BP 217 38043 Grenoble Cedex France; 7 Hospital Universitari de Tarragona Joan XXIII, IISPV, Universitat Rovira i Virgili, Carrer del Doctor Mallafre Guasch 4, 43007 Tarragona, Spain; 8 Department of Heath care, Jönköping County Council, P.O box 1024, SE 551 11 Jönköping, Sweden; 9 Sealed Envelope Ltd., UK; 10 Uppsala Clinical Research, Uppsala University Hospital, SE 751 85 Uppsala, Sweden

## Abstract

**Background:**

Acute coronary syndromes, including myocardial infarction and unstable angina, are important causes of premature mortality, morbidity and hospital admissions. Acute coronary syndromes consume large amounts of health care resources, and have a major negative economic and social impact through days lost at work, support for disability, and coping with the psychological consequences of illness. Several registries have shown that evidence based treatments are under-utilised in this patient population, particularly in high-risk patients. There is evidence that systematic educational programmes can lead to improvement in the management of these patients. Since application of the results of important clinical trials and expert clinical guidelines into clinical practice leads to improved patient care and outcomes, we propose to test a quality improvement programme in a general group of hospitals in Europe.

**Methods/Design:**

This will be a multi-centre cluster-randomised study in 5 European countries: France, Spain, Poland, Italy and the UK. Thirty eight hospitals will be randomised to receive a quality improvement programme or no quality improvement programme. Centres will enter data for all eligible non-ST segment elevation acute coronary syndrome patients admitted to their hospital for a period of approximately 10 months onto the study database and the sample size is estimated at 2,000-4,000 patients. The primary outcome is a composite of eight measures to assess aggregate potential for improvement in the management and treatment of this patient population (risk stratification, early coronary angiography, anticoagulation, beta-blockers, statins, ACE-inhibitors, clopidogrel as a loading dose and at discharge). After the quality improvement programme, each of the eight measures will be compared between the two groups, correcting for cluster effect.

**Discussion:**

If we can demonstrate important improvements in the quality of patient care as a result of a quality improvement programme, this could lead to a greater acceptance that such programmes should be incorporated into routine health training for health professionals and hospital managers.

**Trial registration:**

Clinicaltrials.gov NCT00716430

## Background

Acute coronary syndromes (ACS), including myocardial infarction and unstable angina, are important causes of premature mortality, morbidity and hospital admissions in Europe and worldwide [1,2]. ACS consumes large amounts of health care resources, and has a major negative economic and social impact through days lost at work, support for disability, and coping with the psychological consequences of illness. Given this large health burden it is vital to implement the best cost-effective treatments for ACS.

ACS is usually classified based on the ECG at presentation. Those with persistent ST elevation require an urgent reperfusion strategy with thrombolysis or primary angioplasty, and those without persistent ST-elevation (also called "non-ST elevation") ACS require early risk assessment, intensive medical treatment (including anti-thrombotic and anti-ischaemic drugs), and early revascularisation if clinically indicated. This proposal will focus on the management of patients with non-ST elevation ACS.

The management of patients has to be tailored to individual needs and the availability of resources but it is widely accepted that patients with ACS need high standards of early care as this has a major impact on short and long-term prognosis. Treatments such as aspirin, beta-blockers, heparin and statins should be given routinely to a wide range of patients and for many others clopidogrel and ACE inhibitors are also needed. In addition, invasive procedures such as coronary angiography and revascularisation are becoming more common in an attempt to treat the underlying lesions that may cause ongoing ischaemia and trigger future events [3,4].

Several large registries have shown that there are deficiencies in the treatment of non-ST elevation acute coronary syndromes when compared to recommendations from contemporary guidelines [5-17]. Under-utilisation of evidence-based treatments such as beta-blockers, heparin, statins and ACE inhibitors is common. Recent guidelines recommend targeting more intense treatment to higher risk groups [3,18] but evidence from the registries indicates that, paradoxically, these patients, and particularly subsets of them such as the elderly, diabetics and those with heart failure, often receive less intensive treatment than that recommended [15,17,19,20]. Guidelines also emphasise more intense investigation and treatment including early angiography (within 72 hrs of admission), the use of upstream glycoprotein IIb/IIIa (GP IIb/IIIa) inhibitors and revascularisation, as indicated, especially in higher risk patients. However, the registries again suggest that this strategy is not necessarily targeted at the high-risk patients.

Several models to determine the risk of death, or the composite of death or myocardial infarction (MI) during the in-hospital period and over the ensuing months, have been developed. Some have used data from clinical trials (TIMI, GUSTO, PURSUIT) [21-23] while others have used observational data (NRMI, GRACE) [24,25]. The TIMI and GRACE models [21,25,26] provide a scoring system in which an increased score denotes higher risk and this increases their potential to be used in the routine clinical setting. The ESC guidelines provide a more pragmatic guide by listing features commonly found in high-risk patients, without providing a detailed method of determining risk [3,18].

Improvements in the management of ACS patients can be achieved in a variety of ways. Evidence exists that introducing professional education programmes, care pathways that guide clinical management for common diseases, and audits against guidelines, all improve care [8,10]. There is also evidence that financial incentives can lead to improvements in care, which may take the form of additional payments for achieving pre-defined targets, or as financial losses for failing to do so [27]. In addition, if the overall level of health care funding precludes provision of certain treatments (for example under-provision of coronary revascularisation facilities), this may lead to deficiencies in care that cannot be improved without an increase in health care resources for that particular disease area.

There is growing evidence that the provision of systematic education and training for health professionals can lead to improvements in standards of care (GAP, CRUSADE) [8,10]. Several quality improvement programmes are in progress for ACS patients (GAP, CRUSADE, PROMIS-UK, 'Get-with-the-guidelines') which use recent guidelines (ESC, AHA/ACC) as the basis for the education. The GAP study showed that use of evidence based treatments increased in patients with ST-elevation MI after an education programme was instituted. Rates of use of aspirin and beta-blockers in hospital increased from 81% to 87% (p = 0.02), and 65% to 74% respectively (p = 0.04) after the education programme had been delivered. Improved quality of care has also been shown after education programmes were instituted in the CRUSADE registry of non-ST elevation ACS patients. These studies have shown improvements using historical controls, which has the limitation that the use of evidence based treatments is often increasing anyway over time, and this may confound comparisons between patients enrolled at different times. The GAP programme, along with other studies, has supported the hypothesis that the appropriate provision of simple treatments for ST elevation MI including early thrombolysis, aspirin, beta blockers, statins and ACE Inhibitors are surrogate measures for more general indicators of quality of health care such as organisation of a hospital and motivation of staff.

Preliminary reports are also available for two recent controlled studies of education and quality improvement. The QUICC study [28] used a centre-controlled design to evaluate the efficacy of a quality improvement programme on 5 health care parameters for ACS patients: ACE-inhibitors at discharge (for patients with heart failure or LV dysfunction, diabetes and hypertension), statins (LDL >3.0 mmol/l, total cholesterol >5.0 mmol/l), clopidogrel (non-ST elevation MI), heparin or LMWH in-hospital (non ST-elevation MI) and coronary angiography (non-ST elevation MI with diabetes, prior MI, ST depression, heart failure). 38 hospitals in Sweden which were already participating in the RIKS-HIA registry were allocated to a quality improvement (QI) programme consisting of seminars and workshops to highlight ways to improve care, or to a control group that continued to collect data as before. The RIKS-HIA registry is an ongoing programme where all acute centres in Sweden record core information about all ACS patients using a web-based data collection tool [29,30]. Centres can then interrogate the database to obtain detailed information about their own patients and compare their performance against group data from the other participating hospitals. The QUICC study compared the use of the 5 outcome measures prior to the QI programme and afterwards. There were small increases in the control centres, except for clopidogrel which showed a highly significant increase (presumably because this treatment has recently been approved for use in ACS), but in the QI centres there were highly significant increases in all 5 parameters compared to the changes in the control centres [28].

The PROMIS-UK study used a cluster-randomised design in 38 UK centres. Half the centres received an education programme based on ESC guidelines for the management of non-ST elevation ACS. The primary outcome was the use of aspirin, clopidogrel, beta blockers and statins at discharge, and heparin in-hospital. A total of 1028 patients were enrolled. There was an absolute increase in all of the evidence based treatments between 3.6 to 8.0%. The primary outcome was a composite of all drugs, a score of 1 was allocated for each drug prescribed and the maximum score was 5. Patients managed in the control group had a mean score of 4.12 versus 4.36 (adjusted analysis) for patients in the education programme group, p = 0.048 [31].

Since application of the results of important clinical trials and expert clinical guidelines into clinical practice leads to improved patient care and outcomes, we propose to test a QI programme based largely on the Swedish QUICC experience, in a more general group of hospitals in Europe. If we can demonstrate important changes in quality of patient care, this may lead to a greater acceptance that these programmes should be incorporated into routine training for health professionals and managers.

### Trial hypothesis

The main hypothesis to be tested is that the use of a structured quality improvement programme will lead to measurable improvements in the management of care and use of evidence based treatments for patients presenting to hospital with non-ST elevation acute coronary syndrome.

### Aims of project

a) Assess the efficacy and feasibility of a QI programme for non-ST elevation patients in several European countries

b) Estimate the costs and potential cost-effectiveness of the QI strategy

c) Test the feasibility of using the RIKS-HIA data collection and feedback tool in different European sites and countries

## Methods/study design

This will be a multi-centre cluster-randomised study in 5 European countries: France, Spain, Poland, Italy and UK.

### Selection of centres

EQUIP aims to enrol hospitals representing the full spectrum of care provided to ACS patients. The emphasis however will be on mid-range hospitals that are able to manage the full range of ACS procedures ideally including on-site coronary angiography, but those centres with easy access to coronary angiography at a near-by hospital will also be included. Sites with on-site cardiac surgery will be eligible, with a maximum of two sites permitted per country.

### Study phases

**Figure 1:**
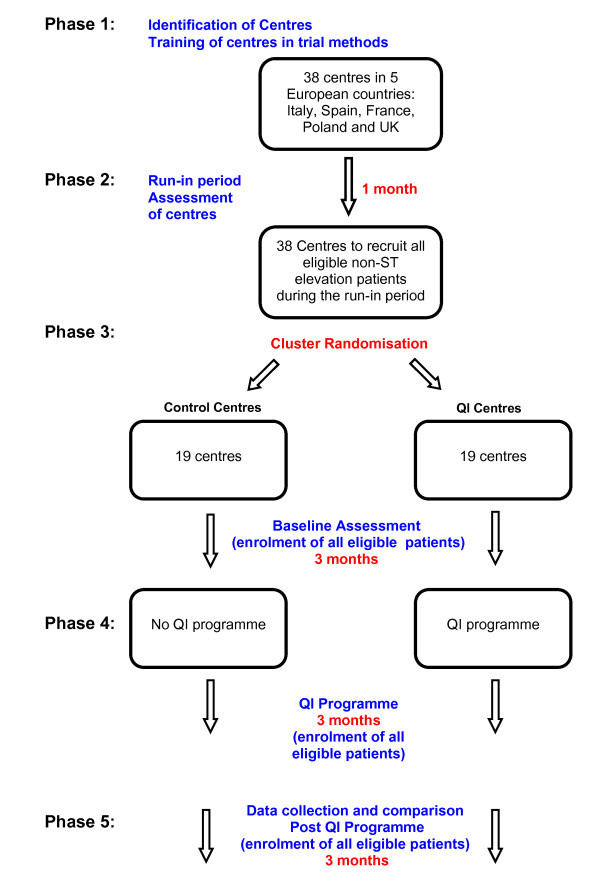
**Flow-diagram**.

The study will be divided into 6 phases as shown in Figure 1.

#### Phase 1

Site selection and set-up phase. Potential centres will be identified by National Co-ordinators and invited to participate. 12 centres will be invited assuming a drop-out rate of 30% leaving on average 8 centres per country. Thus the expectation is that we will have 40 participating centres in total. Centres will then be informed about the study and ethical and regulatory documents obtained.

All participating centres will be provided with a full training programme on the EQUIP project and data collection tool using web-based and telephone training methods.

#### Phase 2

Run-in. Centres will enter a run-in phase for one month to ensure that they are able to collect data properly using the RIKS-HIA web-based system. An assessment of data quality and enthusiasm to participate will be made after this and those centres that are willing and able to continue will be invited to do so.

#### Phase 3

Baseline Phase. Centres will be randomised to either QI programme or control group. Centres will then collect data for 3 months as part of the baseline assessment of care.

#### Phase 4

QI programme (3 months). The main elements of the QI programme will be delivered over a period of 3 months (see **Quality Improvement phase**) during which time all centres will continue to collect data.

#### Phase 5

Centres will collect data for a further 3 months during which the QI centres will receive further input and feedback on performance.

#### Phase 6

Data cleaning, study close-out, statistical analyses and reports

### Patient eligibility

The study aims to enrol patients with a high likelihood of ACS.

### Inclusion criteria

Patients with a good clinical history of ACS and at least one of the following:

a) New or transient ST or T wave changes on the ECG consistent with acute myocardial ischaemia

b) Elevation of troponin or other cardiac markers to levels indicative of myocardial necrosis according to local laboratory values

### Exclusion criteria

• Evidence of persistent ST elevation on the ECG

• Use of early reperfusion therapy (thrombolysis or primary PCI)

• Patients >80 years

• Patients transferred from another hospital

Very elderly patients have been excluded because there is existing evidence that in many cases they are not treated according to guidelines, and thus potential changes in their treatment patterns may not respond to a QI programme. We appreciate that this exclusion criterion is pragmatic and other experts will have differing views on this.

### Outcomes

The outcome measures of this study are consistent with current European guidelines and relate to the management and treatment of non-ST elevation ACS patients.

### Primary outcome

The primary outcome is a composite of eight outcome measures to assess aggregate potential for improvement in care using the QI programme (see statistical section for further discussion). The outcome measures have been developed from the new ESC Guidelines for the diagnosis and treatment of non ST segment elevation acute coronary syndromes [18]. We have only focused on selected recommendations with at least Class I and level of evidence A or B. Text in **bold **indicates direct quotes from the ESC Guidelines. The outcome measures are summarised below:

1. EVIDENCE OF FORMAL DOCUMENTATION OF RISK STRATIFICATION PERFORMED WITHIN 24 HOURS OF ADMISSION

In Section 4.4 of the ESC guidelines it is stated that:

• **Established risk scores (such as GRACE) should be implemented for initial and subsequent risk assessment ***(Recommendation I-B)*

The implementation of initial risk stratification methods will be assessed by this project and this has been interpreted as occurring within the first 24 hours.

Several methods have been shown to help assess risk of patients in the early phase of acute coronary syndrome. The European Society of Cardiology has recently adopted the GRACE model [5,18,25] as part of the European Guidelines and this is the recommended method of risk stratification. A summary of the GRACE model is provided in appendix A.

2. EARLY (<72 HRS) CORONARY ANGIOGRAPHY IN INTERMEDIATE TO HIGH-RISK PATIENTS

In Section 5.4 of the new guidelines:

• **Early (<72 h) coronary angiography followed by revascularisation (PCI or CABG) in patients with intermediate to high-risk features is recommended ***(Recommendation I-A)*

**Definition of intermediate to high-risk patients **(Section 8.3.3 of the guidelines)

The following features indicate patients who should undergo routine early angiography:

• **Elevated troponin levels**

• **Dynamic ST or T wave changes (symptomatic or silent) (≥ 0.5 mm)**

• **Diabetes mellitus**

• **Reduced renal function (GFR<60 mL/min/1.73 m^2^)**

• **Depressed LVEF<40%**

• **Early post-MI angina**

• **PCI within 6 months**

• **Previous CABG**

• **Intermediate to high risk according to a risk score**

For the purposes of this project, ECG changes will be documented as ST-elevation, ST-depression or T wave inversion. In addition, reduced renal function will be defined as estimated creatinine clearance<60 ml/min. It is felt that GFR is not widely established in all hospitals and that calculation of GFR could be impractical in some cases leading to missing information.

Documentation of early post-MI angina and whether a patient has had PCI within 6 months could be difficult and will be omitted for the purposes of this project.

3. ANTICOAGULATION FOR ALL PATIENTS

Section 5.4 of the guidelines:

• **Anticoagulation is recommended for all patients in addition to antiplatelet therapy ***(Recommendation I-A)*

• **Several anticoagulants are available, namely UFH, LMWH, fondaparinux, and bivalirudin. The choice depends on the initial strategy**. *(Recommendation I-B)*

Other more detailed recommendations are provided in the guidelines but for the sake of simplicity UFH, LMWH and fondaparinux would be considered suitable anticoagulants in the early phase of NSTEMI.

4. BETA-BLOCKERS PRESCRIBED AT DISCHARGE IN PATIENTS WITH REDUCED LV FUNCTION

Section 5.5.7 of the new guidelines:

• **Beta-blockers should be given to all patients with reduced LV function ***(Recommendation I-A)*

**Beta blocker therapy should be initiated in all patients and maintained indefinitely in the case of reduced LV function, with or without symptoms of heart failure, unless formal contraindications exist**.

No formal value of LV function is provided by the guidelines. For the purposes of this project, reduced LV function has been defined as LVEF ≤ 50%.

5. STATINS PRESCRIBED WITHIN 4 DAYS OF ADMISSION FOR ALL PATIENTS

In section 5.5.5 of the new guidelines it is stated that

• **Statins are recommended for all NSTE-ACS patients (in the absence of contraindications), irrespective of cholesterol levels, initiated early (within 1-4 days) after admission, with the aim of achieving LDLc levels <100 md/dL (<2.6 mmol/L) ***(Recommendation I-B)*

The second part of this recommendation (achieving LDLc levels <100 md/dL) is beyond the scope of EQUIP.

6. ACE-INHIBITORS AT DISCHARGE IN SELECTED PATIENTS

Section 5.5.8 of the new guidelines:

• **ACE inhibitors are indicated long-term in all patients with LVEF ≤ 40% and in patients with diabetes, hypertension, or chronic kidney disease, unless contraindicated***(Recommendation I-A)*

ARBs prescribed at discharge if ACE-inhibitors are contra-indicated.

The definition of chronic kidney disease will be at the Investigators' discretion and taken from documentation in the medical records.

7. CLOPIDOGREL

A) CLOPIDOGREL LOADING DOSE (≥ 300 MG) ADMINISTERED WITHIN THE FIRST 24 HOURS FROM ADMISSION

Section 5.3.2 of the new guidelines:

**• For all patients, an immediate 300 mg loading dose of clopidogrel is recommended, followed by a 75 mg clopidogrel daily ***(Recommendation I-A)*

For the purposes of EQUIP, we have interpreted 'immediate' as within 24 hours.

B) PRESCRIPTION OF CLOPIDOGREL MAINTENANCE DOSE AT DISCHARGE.

Section 5.3.2 of the new guidelines also recommends:

**• Clopidogrel should be maintained for 12 months unless there is an excessive risk of bleeding**. *(Recommendation I-A)*

The long-term prescription of clopidogrel after discharge is beyond the scope of EQUIP but investigators will be asked to record whether a maintenance dose (75 mg) has been prescribed at discharge.

### Secondary outcomes

a) Clinical outcomes at discharge including death and myocardial infarction

b) Estimated costs of care for patients

c) Estimated costs and economic evaluation of potential cost-effectiveness of QI programme

### Randomisation

EQUIP-ACS has a cluster-randomised study design. Randomisation will be stratified by country and ability to perform percutaneous coronary intervention on-site. Centres will be randomised to the QI programme or not. The co-ordinating centre (CTEU) will inform centres at the beginning of the baseline phase of their randomised allocation. The rationale for this is that personnel at centres allocated to the QI training programme will require sufficient notification to ensure availability and secure travel arrangements for the meetings. There is a possibility that knowledge of the randomised allocation may influence practice patterns. However the influence of this potential bias can be tested by comparing treatment patterns across the two groups during the baseline data collection phase.

### Quality improvement phase

#### QI centres

Centres randomised to the QI programme will receive a "high intensity" quality improvement programme which will be delivered over a three-month period.

From previous experience, especially in the QUICC programme [26], the QI programme should encompass not only guideline driven objectives but also a review of procedures used by centres to manage patients (e.g. bed management, early assessment of patients, responsibilities of different health professionals). Thus the QI programme will have goals and objectives, as well as emphasising process control. It is suggested that at least a senior cardiologist, senior nurse and senior manager attend the QI session from each participating centre. Every effort will be made to standardise the QI intervention and this will be achieved by having the same personnel deliver the programme and for them to work according to a standard operating procedure. This will be contained in the study specific quality improvement manual which will be developed and approved prior to the start of the QI programme. It is important that we compare the QI programme centres to "well managed" control centres. By allowing all centres (QI and control) to use the specialised data collection tool developed by Uppsala we anticipate that any major practice pattern changes will genuinely be the results of the QI programme and not simply because centres have easier access to patient data in an electronic format. Thus the control group will receive a "low-intensity" quality improvement intervention and the QI group will receive a "high intensity" intervention. We believe this reflects "optimum" clinical practice and will allow a fair evaluation of the QI programme.

The following summarises the main elements of the QI programme:

### Preliminary activities

a) Centre to set up a team (a senior cardiologist, a junior cardiologist, senior nurse and senior manager) that have had at least one meeting at their own centre before session 1.

b) The team should be familiar with the EQUIP protocol and contemporary guidelines.

c) The team should be familiar with the registry and the on-line reports. They should review their baseline results before session 1.

d) The team should plan weekly meetings (30-60 minutes) during the phase 4 (QI Programme) period and report their work and results continuously on the EQUIP Internet Portal.

### Detailed structure of QI sessions

#### Session 1 (1 day)

a) Introduction: Why is QI necessary?

b) Short overview of the EQUIP programme

c) Review of the QI programme

d) Goals and methods of measurement

e) Healthcare as a system

f) Identification of potential deficiencies at each centre

g) Centre assignments and contact via Internet portal

#### Session 2 (1 day)

a) Repetition and reflection

b) Report on centre assignments

c) Improvement ideas

d) How to test new ideas

e) Implementation and requirements for sustained improvements

f) Homework (test and implement new ideas, internet portal)

#### Session 3 (1 day)

a) Repetition and review of activities during QI programme

b) Report of tests and results

c) Planning of new tests and/or implementation of successful test

### Control group centres

The centres that are randomised to the control group will receive a "low intensity" programme throughout the 10-month recruitment period. These centres will receive full training on the EQUIP protocol and database (telephone and internet training as for all centres) in addition to access to online reports which will enable them to assess their progress in management of ACS patients.

Regular telephone contact will be maintained with key staff at these centres to discuss general progress, resolve any queries and maintain enthusiasm.

### Patient enrolment

Daily screening of key wards and admission areas will identify potentially eligible patients as soon as possible after admission. All eligible patients will be enrolled into the study during the 10-month study period. Patients will be followed up until hospital discharge only to assess in-hospital treatments and investigations. If a patient is transferred to another hospital for continuing care and will be discharged from there, this transfer will be considered as discharge and staff will be asked to record the reason for transfer (e.g. patient transferred for coronary angiography). Centres will be asked to record data for all eligible patients. Using previous estimates of eligibility and enrolment, we estimate that centres may enrol 10-15 patients per month which translates into 4000-5000 patients in total over a 10-month period. The recruitment period will be approximately 10 months which includes a one-month run-in period, baseline phase (3 months), QI training phase (3 months) and QI implementation phase (3 months). Refer also to Figure 1.

### Data collection

Information about eligible patients will be entered onto the EQUIP-ACS database, which has been adapted from the RIKS-HIA database to include translations into local language and some modifications or additions to fields. The database has a facility to generate online reports which allows centres to assess their progress. The RIKS-HIA data collection tool is a validated web-based system with 10 years of experience [27,28]. All data will be entered in an anonymous manner at the participating centres and transferred in an encrypted format to the main server at UCR, Uppsala where it will be held securely. All data management will take place at UCR.

### Centre training

Before the start of the study, centres will receive training from CTEU and UCR. Centres will be trained on the EQUIP data collection tool using a web-based system and telephone training. The CTEU will provide training and ensure that centres fully understand the protocol and procedures of the study.

### Ethical and regulatory issues

This study is a QI initiative and therefore can be considered part of routine professional activity and education. Except for an increased compliance to accepted standards and professional guidelines, no change to routine health care is expected as part of this study. Ethics Committee Approval has been obtained in each of the 5 countries and for all participating centres for the study to be conducted without obtaining written informed consent from patients. Approvals for data to be transferred from the admission institution to UCR have been obtained from the appropriate local and national authorities. The study will be conducted in accordance with Good Clinical Practice guidelines [32] and the Declaration of Helsinki [33]. Permission and support of local hospital management will be obtained and will be an essential part of the success of this programme. Since this study is not evaluating any specific pharmacological treatment or medical device it falls outside the European Clinical Trials Directive and does not require formal pharmacovigilance.

### Sample size and statistical considerations

The primary outcome is based on the 8 quality improvement variables. Two of the variables will be available in all patients (risk stratification and statin use) whereas the others will be available in a proportion of the whole group. We can document the use of treatments as a proportion at a centre level or at the level of the patient we can score each variable as 1 (administered) or 0 (not administered), allowing a maximum score of 8 for each patient. For the sample size calculation it appeared more straightforward to use the scoring system. Some patients will not contribute to the final analysis as they may not be considered for 5 of the outcome measures. Using a pragmatic assumption we estimate that the average "maximum" score per patient will be 5, allowing for the fact that a proportion will not contribute to the final analysis. To calculate the sample size we have taken the possible differences in score using simulated individual level patient data corrected for centre effect (i.e. that patterns of practice in a particular centre will have similarities across patients treated at that centre and therefore results from each patient cannot be regarded as "independent"). If the maximum score is estimated at 5 each one point would equate approximately to a 20% absolute difference in the frequency of use of that treatment at a centre level. We estimate that a 10% absolute difference would be considered reasonable "minimum" difference to demonstrate that the QI programme can change practice. This equates to an absolute average difference of about 0.5 between the QI centres and control.

In PROMIS-UK [31] we found that a difference of 0.3 between the education group and control group was just detectable with a p value of 0.05, with a total of 1000 patients and 38 centres. The inter-cluster correlation coefficient (ICC) for the control arm of PROMIS was 0.10026.

The sample size estimate for EQUIP uses the following formula:(1)

Where "N_cluster_" and "N_simple_" are the sample sizes for cluster randomisation and simple randomisation respectively. "m" is the number of patients per cluster (i.e. per hospital) and "ICC" is the inter-cluster correlation coefficient.

The table below shows the detectable differences in scores between groups using an ICC of 0.1 or 0.2 and 40 or 50 patients per centre.

Table 1 shows that the study is well powered in the post QI phase to detect differences of about 0.4 with 800 patients assuming an ICC of 0.2. Our anticipated total enrolment over the whole period (baseline, during the QI phase and post QI phase is expected to be a minimum of 2000 and a maximum of 4000, We believe the main "power" of the study will lie in the post QI phase and a total enrolment of 1000 patients (500 per group) will give us good power to detect differences of 0.3-0.4 which equates to absolute differences in rates of use of treatments of 6-8%, Since our minimum clinically important difference is 10%, the study can be considered "well powered". Centres will be asked to recruit all non-ST elevation ACS patients but we do not know the exact enrolment rate in the centres that will take part hence the need to identify a range of sample size estimations. However as this is largely driven by the number of centres it is important we have 40 participating centres.

**Table 1:** Sample size calculation based on score of drug use at discharge

ICC	Centres per Group	Patients per Centre	Patients per Group	SD	Detectable Difference
0.1	20	20	400	0.9	0.312
0.1	20	25	500	0.9	0.302
0.1	20	30	600	0.9	0.295
0.1	20	40	800	0.9	0.286
0.1	20	50	1,000	0.9	0.281
					
0.2	20	20	400	0.9	0.401
0.2	20	25	500	0.9	0.394
0.2	20	30	600	0.9	0.390
0.2	20	40	800	0.9	0.384
0.2	20	50	1,000	0.9	0.380

It was not felt appropriate to use the estimated differences between baseline data and post QI data in the sample size estimation as too many assumptions needed to be made. Therefore we feel we have made a "conservative" sample size estimation that should allow a good opportunity to detect realistic differences between the two groups.

### Statistical analysis plan

The primary outcome is a composite of 8 outcome measures. The proportion of outcomes fulfilled will be calculated on all patients for each time period and group. The change in the proportion over time (Δ = post QI - baseline) will be compared between groups.

Analysis of the primary outcome will be by hierarchical logistic regression to take account of the clustered nature of the data. For each patient up to 8 binary quality indicators will be recorded. The quality indicators are therefore nested within patient. Patients, in turn, are nested within centre. A three-level random effects model will be fitted with random effects for patient and centre. An indicator variable for time period (0 = baseline phase, 1 = post-QI phase) will be entered at level 2 (patient) as a fixed effect and an indicator variable for treatment group (0 = control, 1 = intervention) will be entered as a fixed effect at level 3 (centre). Other centre-level covariates that will be entered as fixed effects at level 3 are country and ability to perform PCI on site (since these will be stratification factors in the randomisation). The interaction between treatment indicator and time period will give a P-value and an estimate of the treatment effect expressed as an odds ratio for the probability of a quality indicator being fulfilled.

### Trial organisation and committees

#### Study sponsor

The Royal Brompton and Harefield Trust will act as the Sponsor of this study. The Sponsor's role is clearly defined in the ICH Good Clinical Practice guidelines [32]. Research agreements will be held with the participating centres.

#### Steering committee

The Steering Committee (SC) is responsible for maintaining the scientific integrity and supervising the progress of the study. The SC will approve the study protocol and any subsequent amendments. The SC will meet prior to the start of the study and as required for the duration of the study.

#### National co-ordinators

The National Co-ordinators (NCs) will be responsible for identification of suitable centres in their country and maintaining contact with these, in addition to liaising with the central co-ordinating centre (CTEU) and the data management centre (UCR) to resolve any issues arising. The NCs will be responsible for obtaining national regulatory, ethical and local institutional site approval for the study in addition to ensuring approval of all subsequent amendments to the study. The NCs will assist with site training and the QI programme meetings.

#### Investigators

It is the responsibility of the Investigator at each participating site to ensure that approval is obtained from the appropriate local ethics committee and that a formal Agreement is signed by the appropriate site signatory. Investigators will be expected to ensure compliance with the protocol and all study documentation and to perform the study in accordance with Good Clinical Practice and the Declaration of Helsinki.

The Local Principal Investigators will be required to identify a research team to assist with the EQUIP study. Investigators are required to allow access to study documentation or source data on request for monitoring visits and audits performed by the CTEU or any regulatory authorities.

Each Investigator and members of the local research team are responsible for the accuracy, completeness and legibility of the data entered onto the EQUIP database and all associated reports. The Investigator must keep a list containing all patients enrolled into the study. This patient list remains with the Investigator and is used for unambiguous identification of each patient. The list contains the patient identification numbers, full names, dates of birth and dates of enrolment in the study. These data should identify the trial and should document the dates of the patient's participation.

The Investigator will preserve all records associated with the study for 10 years or for a period to be determined by the coordinating centre.

#### Data management

Uppsala Clinical Research (UCR) will be responsible for the data collection and management of the study. UCR will provide the EQUIP database and Internet Portal and contribute to the QI meetings in addition to assisting with site training.

#### Study co-ordination

The study will be co-ordinated and managed by the Clinical Trials and Evaluation Unit (CTEU) at the Royal Brompton Hospital, London UK. The CTEU will assist in preparing the final protocol, the investigators' Manual of Operations and will assist in training centre staff on the RIKS-HIA database (telephone and web training) in addition to participating and assisting in the QI meetings.

#### Publication policy and dissemination of results

The results of the study will be submitted for publication to a peer-reviewed journal irrespective of the outcome. The Steering Committee will be responsible for approval of all manuscripts arising from the study prior to submission for publication. Sub-studies of centre-specific data may only be carried out with the knowledge and approval of the Steering Committee.

### End of trial

#### Planned termination

The trial will end when all patients have completed the observation period, the database has been declared clean and the main results have been analysed and submitted for publication.

## Discussion

If we can demonstrate important improvements in the quality of patient care as a result of a quality improvement programme, this could lead to a greater acceptance that these programmes should be incorporated into routine health training for health professionals and hospital managers.

## List of abbreviations used

ACS: Acute Coronary Syndromes; CABG: Coronary Artery Bypass Grafting; CTEU: Clinical Trials and Evaluation Unit; ECG: Electrocardiogram; EQUIP: European Quality Improvement Programme for Acute Coronary Syndromes; GFR: Glomerular Filtration rate; ICC: Inter-cluster Correlation Coefficient; ICH: International Conference on Harmonisation; LVEF: Left Ventricular Ejection Fraction; LMWH: Low Molecular Weight Heparin; MI: Myocardial Infarction; NC: National Co-ordinator; PCI: Percutaneous Coronary Intervention; QI: Quality Improvement; SC: Steering Committee; UCR: Uppsala Clinical Research; UFH: Unfractionated heparin.

## Competing interests

MF: Has received consultancy fees from GSK and research grant funding from GSK to his institution to co-ordinate this project. HB: Has received honoraria from GSK. PGS: Has received speakers' and consultancy fees from GSK. All other authors confirm that they have no competing interests to declare.

## Authors' contributions

MF: participated in the design of the study and helped to draft the manuscript. JB: participated in the design and co-ordination of the study and helped to draft the manuscript. DB: participated in the design and co-ordination of the study and helped to draft the manuscript. HB: participated in the design of the study and helped to draft the manuscript. PGS: participated in the design of the study and helped to draft the manuscript. GO: participated in the design of the study and helped to draft the manuscript. FO: participated in the design of the study and helped to draft the manuscript. JM: participated in the design of the study and helped to draft the manuscript. AB: participated in the design of the study and helped to draft the manuscript. MB: contributed to the design of the Quality Improvement programme.

ARB: developed the statistical analysis plan. BL: participated in the design of the study and helped to draft the manuscript.

## Appendix A

### Summary of GRACE Model

Three risk categories have been developed from the GRACE risk model and these are summarised in the Tables 2 and 3 for in-hospital and six-month mortality.

**Table 2:** Mortality in hospital

Risk Category (tertiles)	GRACE Risk Score	Probability of death in-hospital (%)
**Low**	1-108	<1

**Intermediate**	109-140	1-3

**High**	141-372	>3

**Table 3:** Mortality at Six months

Risk Category (tertiles)	GRACE Risk Score	Probability of death post-discharge to 6 months (%)
**Low**	1-88	<3

**Intermediate**	89-118	3-8

**High**	119-263	>8
